# Predictors of discontinuing exclusive breastfeeding before six months among mothers in Kinshasa: a prospective study

**DOI:** 10.1186/s13006-015-0044-7

**Published:** 2015-05-27

**Authors:** Pélagie Babakazo, Philippe Donnen, Pierre Akilimali, Nathalis Mapatano Mala Ali, Emile Okitolonda

**Affiliations:** Kinshasa School of Public Health, University of Kinshasa, Kinshasa, Democratic Republic of the Congo; School of Public Health, Université Libre de Bruxelles, Brussels, Belgium

**Keywords:** Exclusive breastfeeding, Predictors, Democratic Republic of the Congo

## Abstract

**Background:**

Although breastfeeding is common in Democratic Republic of the Congo, the proportion of women who exclusively breastfeed their babies up to 6 months remains low. This study aimed at identifying predictors of discontinuing exclusive breastfeeding before six months among mothers in Kinshasa.

**Methods:**

A prospective study was carried out from October 2012 to July 2013 among 422 mother-child pairs recruited shortly after discharge from twelve maternities in Kinshasa and followed up to six months. Interviews were conducted at each woman’s house during the first week after birth, and at one, two, three, four, five and six months. Collected data included history of child’s feeding and mother’s socio-demographic and psychosocial characteristics. The Cox Proportional Model was used to identify predictors of discontinuing exclusive breastfeeding before six months.

**Results:**

The median duration of exclusive breastfeeding was 10.9 weeks (Inter Quartile Range 4.3 to 14.9). At six months, 2.8 % of infants were exclusively breastfed. The factors independently associated with the discontinuation of exclusive breastfeeding before six months were: not confident in the ability to breastfeed [Adjusted hazard ratio (AHR) = 3.90; 95 % CI 1.66, 9.16)], no plan on the duration of EBF (AHR = 2.86; 95 % CI 1.91, 4.28), breastfeeding problems during the first week (AHR = 1.54; 95 % CI 1.13, 2.11), low level of breastfeeding knowledge (AHR = 1.52; 95 % CI 1.08, 2.15), and experienced less than five Baby-friendly practices during the maternity stay (AHR = 1.47; 95 % CI 1.05, 2.06).

**Conclusions:**

Confidence in the ability to breastfeed and intention to exclusively breastfeed were the most important predictors of discontinuing exclusive breastfeeding before six months. To have a greater impact on the duration of exclusive breastfeeding, interventions should focus on these factors.

**Electronic supplementary material:**

The online version of this article (doi:10.1186/s13006-015-0044-7) contains supplementary material, which is available to authorized users.

## Background

Much of a child’s future is determined by the quality of nutrition in the first 1,000 days, from conception to 24 months of age. When the child’s brain and body are developing rapidly, good nutrition is essential to lay the foundation for a healthy and productive future. The World Health Organization (WHO) recommends exclusive breastfeeding (EBF) for the first six months, followed by timely, adequate, safe and appropriate complementary feeding, while continuing breastfeeding for two years and beyond [1]. Breastfeeding has many benefits for infants, mothers and families. Breastfeeding ensures child’s growth, protects against common acute childhood infections [2, 3], decreases the rate of Sudden Infant Death Syndrome [4], promotes cognitive development and, prevents atopic diseases, obesity and diabetes mellitus [5]. For the mother, breastfeeding is economic and ensures maternal-child bonding, postpartum weight loss and birth spacing. Furthermore, breastfeeding is protective against breast and ovarian cancers [2].

The Lancet series on child survival estimated that, in low-income countries, 13 % of deaths among children under-five could be prevented if breastfeeding prevalence were increased to the optimal coverage of 90 % [6]. In 2010, among children younger than five years, suboptimal breastfeeding was responsible for 8 % of all deaths and 7.6 % of all childhood Disability Adjusted Life Years (DALYs) [7]. Large decreases in deaths and DALYs attributable to suboptimal breastfeeding were noted from 1990 to 2010. However, 5 % of developing countries, including the Democratic Republic of the Congo (DRC), had an increase in absolute number of DALYs attributable to suboptimal breastfeeding [7]. Half of all deaths of under-five children occur in just five countries: India, Nigeria, Pakistan, DRC and China [8]. In DRC, no significant progress has been noted towards reaching the Millennium Development Goal (MDG) related to the reduction of child mortality, MDG 4 [9]. Although breastfeeding is common, the proportion of women who exclusively breastfeed their babies up to six months remains low. During the Demographic and Health Survey (DHS) carried out in 2007, only 36 % of children younger than six months were exclusively breastfed [10]. To promote the practice of EBF, the country had implemented various strategies including the extension of the Baby Friendly Hospital Initiative (BFHI) in health facilities located in provinces, the establishment of Baby Friendly Communities and the implementation of the national code of the marketing of breast milk substitutes. In spite of those interventions, no significant change was noted in 2010. Indeed, the result from the 2010 Multiple Indicator Cluster Surveys (MICS) showed that nearly all mothers initiated breastfeeding, 37 % of children younger than six months were exclusively breastfed, 65 % of children from two to three months old were receiving complementary feeding and, only 5 % of children were exclusively breastfed at six months [11].

To set up successful breastfeeding promotion strategies, understanding the determinants of breastfeeding duration is necessary. A large number of studies carried out to evaluate socio-demographic factors associated with breastfeeding duration reported that women who are older, married, better educated and of higher income breastfeed longer [12–16]. Socio-demographic factors are almost non-modifiable by midwife interventions. To have an impact on breastfeeding duration and exclusivity, interventions should focus on modifiable factors. The modifiable factors that are positively associated with breastfeeding duration are the woman’s intention to breastfeed and breastfeeding self-efficacy, and the social support [12, 17]. Other factors associated with breastfeeding duration are hospital practices [18–20]. The reference for the improvement of breastfeeding practices in health facilities is the BFHI, summarized in the “Ten Steps to Successful Breastfeeding”: (1) Having a written breastfeeding policy that is routinely communicated to all health care staff, (2) training all health care staff in skills necessary to implement this policy, (3) informing all pregnant women about the benefits and management of breastfeeding, (4) helping mothers to initiate breastfeeding within a half-hour of birth, (5) showing mothers how to breastfeed and how to maintain lactation even if they should be separated from their infants, (6) giving newborn infants no food or drink other than breast milk unless medically indicated, (7) allowing mothers and infants to remain together 24 h a day, (8) encouraging breastfeeding on demand, (9) giving no artificial teats or pacifiers to breastfeeding infants and, (10) fostering the establishment of breastfeeding support groups and referring mothers to them on discharge from the hospital or clinic. A health facility providing maternity services and care for newborn infants can be awarded the “Baby-Friendly” designation if it has implemented the Ten Steps and met the assessment criteria of the national Baby-Friendly authority.

In Kinshasa, capital of DRC, only seven health facilities had been designed “Baby-Friendly”. Yotebieng had reported mother’s reasons for starting supplementation with liquid, formula and porridge before six months in this setting [21]. However, to our knowledge, no study has been published on predictors of EBF duration, including baby-friendly practices. Due to socio-cultural differences, the factors identified elsewhere may or may not be the same in Kinshasa. This study aimed to identify predictors of discontinuing EBF before six months among mothers in Kinshasa.

## Methods

### Study design and setting

A prospective study on infant feeding practices was carried out among mother-child pairs in Kinshasa from October 2012 to July 2013. Mothers were recruited during the first week after childbirth and followed for six months.

### Sampling procedure

Kinshasa has six health districts, two (Ndjili and Kalamu) were randomly selected for the study. The list of all maternity facilities located in these two selected health districts and the monthly mean number of births occurred in each maternity facility were obtained from the National Reproductive Health Program. From this list, 12 eligible maternity facilities were randomly selected. To be eligible for the study, at least 60 births should occur in the maternity facility per month. In the selected maternity facility, all eligible women who gave birth from October 2012 to December 2012 were recruited. The mother was considered to be eligible when she was at least 18 years old, had antenatal care visits in the same maternity facility and gave birth to a single living full-term child who was free of any serious health conditions that would require transfer to an intensive care unit.

### Data collection

To collect data, six trained surveyors led face-to-face interviews at seven times: during the first week after birth, and at 1, 2, 3, 4, 5, and 6 months. Those interviews were conducted at the mother’s home to allow her to feel free while talking about what she had experienced in the health facility and how she was feeding her infant.

Twice a week, surveyors identified eligible mothers in the registers of the selected maternity facilities. Then, mothers were contacted at home during the first week after birth; they were informed about the study and its purpose. Those agreeing to participate gave written consent. During this first visit, the mother’s socioeconomic, psychosocial and medical data were collected. She was also asked about baby-friendly practices experienced during the stay in the maternity and difficulties related to breastfeeding encountered. During interviews led at 1, 2, 3, 4, 5 and 6 months, mothers were asked about how their infants were fed the previous day. If any food or drink other than breast milk was mentioned, the mother was asked about when she started to feed her infant with the food or drink in question. The study instrument was translated in Lingala and back in French, the original language.

### Definition of variables

The breastfeeding terms used in this study were those recommended by the WHO. An infant was considered to be exclusively breastfed when he or she had received only breast milk with no other liquids (including water) or solids [22]. The duration of EBF was determined using information about the age at which other types of milks, liquids and/or complementary foods were introduced. This duration was initially measured in days, and then converted in weeks.

The mother’s intention to breastfeed exclusively was defined as the planned length of EBF. The Breastfeeding Self-Efficacy Short Form (BSE-SF) was used to assess the mother’s confidence in her ability to breastfeed [23], while the attitude toward breastfeeding was evaluated with the lowa Infant Feeding Attitude Scale (IFAS) [24]. A cross-cultural adaptation was made from the original scales; items that were not culturally adapted to Kinshasa population were removed. Thus, 10 items from each original scale were used in this study. Each item was measured on a 5-point scale, and the total score ranged from 5 to 50. For the purpose of bivariate and multivariate analysis, confidence in the ability to breastfeed was split into three groups: not confident (less than 35), fairly confident (from 35 to 44) and very confident (at least 45). For the same purpose, the attitude toward breastfeeding was also split into three groups: negative attitude (less than 35), fairly positive attitude (from 35 to 44) and very positive attitude (at least 45). In absence of a validated standardized questionnaire, the mother’s knowledge on breastfeeding was determined with a pretested questionnaire adapted from previous studies [25, 26]. This questionnaire included ten questions related to the following aspects: timing of breastfeeding initiation, importance of colostrum, average number of feeds a child should receive per day, duration of EBF and mother’s understanding of the usefulness of breastfeeding. Each question was given a rating of 0 or 1 depending on whether the answer was wrong or correct. Therefore, the total score ranged from 0 to 10. For the purpose mentioned above, the knowledge on breastfeeding was split into three groups: low level of knowledge (less than 5), fairly good level of knowledge (from 5 to 6) and very good level of knowledge (at least 7).

Ten questions were used to assess baby-friendly practices experienced by mothers during the stay in the maternity (see Additional file 1). Thus, this variable ranged from 0 to 10. These questions covered six of the ten steps to successful breastfeeding namely: informing all pregnant women about the benefits and management of breastfeeding (step 3), helping mothers to initiate breastfeeding within a half-hour of birth (step 4), giving newborn infants no food or drink other than breast milk, unless medically indicated (step 6), allowing mothers and infants to remain together 24 h a day (step 7), encouraging breastfeeding on demand (step 8) and giving no artificial teats or pacifiers to breastfed infants (step 9).

The family income was defined as the average amount spent by the family, per day and per individual, for foods. The family was considered to have a low income when this amount was less than one United States Dollars (1 USD).

The questionnaire used to collect data was assessed for reliability using the test-retest method. Twenty breastfeeding mothers were interviewed, and then re-interviewed after seven days. The correlation coefficient ranged from 0.84 to 0.96.

### Data analysis

Information collected was entered in EpiData version 3.1 and analyzed with Stata version 12. Characteristics of study participants were summarized using the median and interquartile range (IQR) for continuous variables and proportions for categorical variables namely: occupation, education, marital status and parity.

Survival analysis was used to examine the duration of EBF. For this analysis, the duration of EBF was right censored at the age of the child at the moment the mother-infant pair had dropped out of the study and at six months for mothers who continued to exclusively breastfeed at the end of the study. The child death was also considered as a censoring event. The Kaplan Meier Method was used to determine the median duration of EBF. The Cox Proportional Model helped to identify predictors of discontinuing EBF before six months. Crude hazard ratios (CHR) and adjusted hazard ratios (AHR) had been calculated in bivariate and multivariate analysis respectively. The following variables were included in the model, which was built using the stepwise approach: age, education level, marital status, occupation, family income, parity, number of antenatal care visits reached, intention to breastfeed exclusively, confidence in the ability to breastfeed, attitude toward breastfeeding, knowledge on breastfeeding, breastfeeding problems during the first week and number of BFHI practices experienced. The final model was built with six variables namely: number of antenatal care visits reached, intention to breastfeed exclusively, confidence in the ability to breastfeed, knowledge on breastfeeding, breastfeeding problems during the first week and number of BFHI practices experienced. The compliance of the model with the assumption of proportionality was assessed using the plot based on Schoenfeld residues. Statistical significance was set at *p* < 0.05.

### Ethical consideration

The study was approved by the Ethical Committee of the Kinshasa School of Public Health. At the time of recruitment, all mothers were informed about the study and provided a signed informed consent. Participation was entirely voluntary and mothers had the liberty of withdrawing from the study at any time.

## Results

**Fig. 1:**
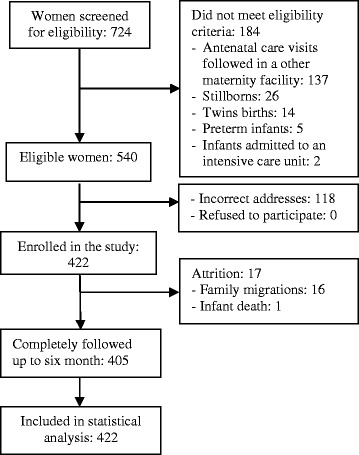
Participants flow chart

A total of 422 mother-child pairs were recruited for the study and, 405 (96.0 %) were followed for the full six months. Attrition was due to the migration of the family (16/17) or to the child’s death (1/17). All 422 recruited mothers were included in the statistical analyses. Flow of participants from recruitment to statistical analysis is presented in Fig. 1.

 Table 1 shows socio-demographic characteristics of participants. Almost three fifths of participants were 20 to 29 years old. The median age of the mothers was 26 years (IQR 22 to 31). Nearly half of the mothers (49.1 %) had completed primary education. Most mothers (85.3 %) were married or living with a boyfriend. Regarding the family income, most families of participants (72.5 %) had expended at least 1USD per inhabitant and per day. Three fifths of participants had ever been mother.

**Table 1:** Characteristics of study participants

	Number n = 422	Percent
Age (years)		
<20	47	11.2
20-29	241	57.2
≥30	133	31.6
Education level		
Never been at school	8	1.9
Primary	207	49.1
Secondary	179	42.4
University	28	6.6
Marital status		
Living with a partner	360	85.3
Single	62	14.7
Occupation		
Housewife	204	48.3
Small trade	106	25.1
Hairdresser/dressmaker	68	16.1
Paid job	20	4.7
Student	18	4.3
Farm worker	6	1.4
Family income		
≥1$ per habitant	264	72.5
<1$ per habitant	100	27.5
Parity		
1	162	38.4
2-3	167	39.6
≥4	93	22.0

All 422 mothers initiated breastfeeding, 233 (55.2 %) did it within an hour of birth and 73 (17.3 %) within thirty minutes. During the maternity stay, 369 (87.5 %) children were exclusively breastfed. At six months, only 12 (2.8 %) infants were exclusively breastfed. The highest rates of EBF discontinuation were noticed during the first and fourth months. Twenty-five percent of mothers stopped EBF during the first month, 17 % during the second month, 18 % during the third month and 22 % during the fourth month. The median duration of exclusive breastfeeding was 10.9 weeks (IQR 4.3 to 14.9).

Regarding predictors of EBF up to six months, none of the mother’s socio-demographic characteristics was associated with the discontinuation of EBF (Table 2). The factors significantly associated with the discontinuation of EBF in bivariate analysis, were the following: having reached less than four antenatal care visits, no plan on the duration of EBF, lack of confidence in the ability to breastfeed, low level of knowledge on breastfeeding, breastfeeding problems during the first week, and having experienced less than five BFHI practices during the stay in the maternity facility. The mother’s attitude toward breastfeeding was not associated with EBF up to six months (Table 2).

**Table 2:** Factors associated with the discontinuation of exclusive breastfeeding in bivariate analysis

Variable	n	Persons-weeks	Events	CHR^a^ (95 % CI)	*p*-value
Age (years)					
<20	47	476.57	45	1.15 (0.82, 1.62)	0.424
20-29	241	2459.86	229	1.05 (0.84, 1.30)	0.671
≥30	133	1438.86	127	1	
Education level					
At most primary	215	2193.57	207	1.13 (0.93, 1.37)	0.231
At least secondary	207	2188.14	195	1	
Marital status					
Living with a partner	62	605.86	61	1.28 (0.97, 1.68)	0.081
Single	360	3775.86	341	1	
Occupation					
Work outside the home	218	2245.29	208	1.05 (0.86, 1.27)	0.650
Housewife	204	2136.43	194	1	
Family income					
≥1$ per habitant	264	2664.71	252	1.11 (0.88, 1.41)	0.377
<1$ per habitant	100	1118.43	96	1	
Parity					
1	162	1571.00	156	1.04 (0.81, 1.35]	0.754
2-3	167	1842.57	155	0.82 (0.63, 1.06)	0.134
≥4	93	968.14	91	1	
Number of ANC^b^ visits reached					
<4	137	1284.71	133	1.41 (1.14, 1.74)	0.002
≥4	284	3090.86	268	1	
Planned length of EBF					
Nothing planned	39	228.29	39	2.75 (1.92, 3.92)	<0.001
<6 months	186	1754.18	179	1.44 (1.17, 1.78)	0.001
≥6 months	190	2331.29	178	1	
Confidence in the ability to breastfeed					
Not confident	9	49.86	9	4.68 (2.34, 9.34)	<0.001
Fairly confident	266	2483.00	260	1.96 (1.57, 2.44)	<0.001
Very confident	146	1848.86	132	1	
Attitude toward breastfeeding					
Negative attitude	72	696.00	71	1.41(0.98, 2.03)	0.067
Fairly positive attitude	292	3001.14	282	1.35 (0.99, 1.84)	0.056
Very positive attitude	55	629.29	48	1	
Knowledge on breastfeeding					
Low level	60	401.14	57	1.95 (1.45, 2.62)	<0.001
Fairly good level	156	1450.14	151	1.47 (1.18, 1.82)	<0.001
Very good level	205	2504.71	194	1	
Breastfeeding problems during the first week					
Yes	72	534.29	70	1.63 (1.26, 2.11)	<0.001
No	350	3847.43	332	1	
Number of BFHI ^c^ practices experienced					
<5	64	524.86	62	1.55 (1.15, 2.09)	0.004
5 - 6	193	1850.00	186	1.45 (1.16, 1.80)	0.001
≥7	157	1940.86	146	1	

^a^Crude Hazard Ratio, ^b^Antenatal care, ^c^Baby-Friendly Hospital Initiative

Regarding each baby-friendly hospital practice individually, mothers who had exclusively breastfed during the stay in the maternity (CHR = 1.59; 95 % CI 2.14, 1.18), those who had been helped to breastfeed correctly (CHR = 1.55; 95 % CI 1.25, 1.92)] and those who were informed about the benefits of breastfeeding (CHR = 1.32; 95 % CI 1.08, 1.62), had higher hazard of EBF at six months. Initiating breastfeeding within 30 min after birth was not significantly associated with the discontinuation of EBF (CHR = 1.28 (95 % CI 0.99, 1.66).

In multivariate analysis, confidence in the ability to breastfeed was the strongest factor associated with discontinuing EBF before six months; the second was the intention to exclusively breastfeed (Table 3). The hazard of not being exclusively breastfeed at six months was four times and three times higher respectively among mothers who were not confident in their ability to breastfeed and mothers who had no plan about the duration of EBF. Other factors independently associated with the discontinuation of EBF were the following: breastfeeding problems during the first week (AHR = 1.54 (95 % CI 1.13, 2.11), low level of knowledge on breastfeeding (AHR = 1.52; 95 % CI 1.08, 2.15) and experienced less than five BFHI practices during the maternity stay (AHR = 1.47; 95 % CI 1.05, 2.06) (Table 3).

**Table 3:** Factors associated with the discontinuation of exclusive breastfeeding in multivariate analysis (n = 409)

Variable	AHR^a^ (95 % CI)	*p*-value
Number of ANC^b^ visits		
<4	1.27 (1.00, 1.62)	0.054
≥4	1	
Planned length of EBF		
Nothing planned	2.86 (1.91, 4.28)	<0.001
<6 months	1.29 (1.01, 1.64)	0.039
≥6 months	1	
Confidence in the ability to breastfeed		
Not confident	3.90 (1.66, 9.16)	0.002
Fairly confident	1.53 (1.19, 1.97)	0.001
Very confident	1	
Knowledge on breastfeeding		
Low level	1.52 (1.08, 2.15)	0.018
Fairly good level	1.30 (1.01, 1.68)	0.040
Very good level	1	
Breastfeeding problems during the first week		
Yes	1.54 (1.13, 2.11)	0.006
No	1	
Number of BFHI^c^ practices experienced		
<5	1.47 (1.05, 2.06)	0.026
5 - 6	1.33 (1.04, 1.70)	0.023
≥7	1	

^a^Adjusted Hazard Ratio, ^b^Antenatal care, ^c^Baby-Friendly Hospital Initiative

The study power was estimated at 1.00 using Stata 12 with the following parameters: sample size: 422, probability of failure observed: 0.9597 (1–17/422), and default values of Hazard ratio (HR), standard deviation (SD) and type of test.

## Discussion

This study aimed to identify predictors of discontinuing EBF before six months among mothers in Kinshasa. The results showed a low rate of exclusive breastfeeding at six months (2.8 %), and the main predictors of discontinuing EBF before six months were the lack of confidence in the ability to breastfeed and having no plan about the duration of EBF.

Rate of EBF at six months during this study is almost similar to the rate reported in Kenya (2 %) [16]. These findings suggest that very few women breastfeed exclusively up to six months in Africa, although in this part of the world, almost all mothers initiate breastfeeding. Interventions have to be set up to increase the rate of children exclusively breastfed up to six months.

In this study, the highest rates of EBF discontinuation were noticed during the first and fourth months. Breastfeeding problems encountered during the first days after childbirth could explain the high rate of EBF discontinuation during the first month of life. The high number of mothers who stopped exclusive breastfeeding during the fourth month was probably due to the resumption of income-generating activities. In DRC and in particular in Kinshasa, when a woman gives birth, she stays at home for around three months in order to take care of the baby and ensure her recovery. Then, she resumes her routine activities.

In this cohort, less than two thirds of mothers (55.2 %) initiated breastfeeding within an hour of birth, similar findings were reported in Kenya [16]. Regarding the exclusivity of breastfeeding, the majority (87.5 %) of children were exclusively breastfed at the maternity discharge. This rate decreased to 75 %, 50 % and 25 % at four, eleven and fifteen weeks of life respectively. These findings suggest that, the antenatal support received by mothers is not sufficient in itself to increase the duration of EBF. Therefore, it should be followed by a postnatal breastfeeding support to help mothers to overcome breastfeeding challenges.

A large literature on socio-demographic factors associated with breastfeeding duration and exclusivity widely acknowledge that women who are younger, less educated, single, of lower income, unemployed and first-time mother, breastfeed shorter [13–16, 27]. Similar to other studies [28, 29], the current study did not find association between EBF up to six months and the following mother’s socio-demographic characteristics: age, level of education, marital status, family income, occupation, and parity. Given that the study had sufficient power (1.00), this finding can be explained by the fact that the association between these factors and breastfeeding practice is influenced by others factors. The effect of the marital status on breastfeeding duration, for instance, may be influenced by the father’s attitude toward breastfeeding. A woman whose partner has a negative attitude toward breastfeeding may breastfeed for shorter time periods. Regarding the family income, in low-income countries, women with low economic status may have less access to complementary foods and breast-milk substitutes. Thus, EBF may be the only option available to them. Finally, the effect of the mother’s employment on breastfeeding depends on the job flexibility. Full-time employees stop earlier breastfeeding than unemployed mothers. However, self-employed mothers did not differ significantly from unemployed mothers regarding their breastfeeding practice [30].

Psychosocial factors seems to be the most important modifiable predictors of breastfeeding duration [12, 31]. In this study, the strongest predictor of discontinuing EBF before six months was the lack of confidence in the ability to breastfeed. The second predictor in importance was being undecided on the expected duration of EBF. These two predictors are highly correlated; the breastfeeding self-efficacy predicts the intention to breastfeed [31]. Interventions to promote EBF should focus on the improvement of the mother’s breastfeeding self-efficacy. This improvement could be achieved by watching other mothers breastfeeding, by the mothers’ own successful breastfeeding experiences, by her perception of being supported by people in her social network (including health professionals, friends and, the child’s father and grandmother), and by a healthy physical and mental status [32].

According to this study, mothers who had a low level of knowledge on breastfeeding had lower hazard of EBF at six months. The same observation was made by other authors in Sub-Saharan Africa countries [13, 33]. There was no significant relationship between cessation of EBF before six months and the mother’s attitude toward breastfeeding. This result, in contradiction with the literature [27], can be explained by the fact that the lowa Infant Feeding Attitude Scale assess the mother’s attitude toward breastfeeding in opposition to formula. Another instrument should be developed and validated for the assessment of the attitude toward EBF.

Mothers who encountered breastfeeding problems during the first week were more likely to stop EBF before six months. The mother’s confidence in her ability to breastfeed, even though high during the antenatal period, can suddenly decrease early in the postpartum period, if she encounters any difficulty to breastfeed. On the other hand, a mother who encountered problems and who overcome them develops greater breastfeeding self-efficacy. During breastfeeding education sessions, role-play activities on solving breastfeeding problems may be a useful strategy to increase the mother’s breastfeeding self-efficacy.

In this study, the more the mother experienced BFHI practices, the longer she exclusively breastfed. The BFHI is the global initiative of WHO and UNICEF that gives to every baby the best start in life by creating a health care environment that protects, promotes and supports breastfeeding. Internationally, impact studies have demonstrated that, if well integrated, the initiative is an effective intervention to increase breastfeeding initiation, duration, and exclusivity [19, 20, 34]. Findings of this study reinforce this evidence and may suggest the extension of the BFHI to all Kinshasa maternity facilities. Contrary to the literature [12], the timing of breastfeeding initiation was not associated with EBF duration. This contradiction could be due to the fact that, this variable, measured retrospectively, might be incorrectly estimated by the mother.

This study can be interpreted in light of its strengths and limitations. One of the strengths of this study is that the low reliability of EBF duration determined retrospectively was avoided. The prospective approach was chosen to better understand the breastfeeding practice over time. In addition, the study had sufficient power and less than 5 % of lost to follow-up were noted. With regard to the limitations, the recall bias was not completely avoided. Indeed, the day when foods or drinks other than breast milk were introduced was determined retrospectively. Therefore, mothers could or could not remember precisely that moment.

## Conclusions

In this study, very few mothers exclusively breastfed up to six months, regardless of age, education, occupation, marital status, family income and parity. The main determinants of EBF duration include confidence in the ability to breastfeed and the intention to exclusively breastfeed. BFHI practices were also found to have an impact on EBF up to six months; the more a mother experienced BFHI practices the longer she was exclusively breastfeeding. To promote successful EBF among mothers in Kinshasa, interventions should focus on the improvement of the mother’s confidence in her ability to breastfeed and on the extension of the BFHI to all maternity facilities.

## Additional file

Additional file 1:
**Questions used to inquire about exposure to baby-friendly practices.**

## Abbreviations

AHZ: Adjusted hazard ratio
BFHI: Baby-friendly hospital initiative
CHR: Crude hazard ratio
DRC: Democratic Republic of the Congo
EBF: Exclusive breastfeeding
IQR: Interquartile range
