# Supplementary material for: Predictors of discontinuing exclusive breastfeeding before six months among mothers in Kinshasa: a prospective study
Source: Int Breastfeed J. 2015 May 27;10:19. doi: 10.1186/s13006-015-0044-7 (PMC4464639; doi:10.1186/s13006-015-0044-7)
Supplement: Additional file 1: — Questions used to inquire about exposure to baby-friendly practices. [file 13006_2015_44_MOESM1_ESM.doc]

**Questions used to inquire about exposure to baby-friendly practices**

| In the maternity facility where you had antenatal care visits and you gave birth:   1. Were you informed about breastfeeding benefits? 2. Did you and your baby benefit from skin-to-skin contact immediately after birth? 3. After the child birth, how time it elapsed before you started breastfeeding? 4. Did health care providers help you to initiate breastfeeding within thirty minutes after birth? 5. Did they seek to watch at least one of your baby feedings? 6. If needed, did they help you to learn to position and attach your baby? 7. Were foods or drinks other than breast milk given to your baby? 8. Did you remain together with your baby 24 hours a day? 9. Did heath care providers encourage you to breastfeed on demand? 10. Were artificial teats or pacifiers given to your baby? |
| --- |
